# Laboratory validation of patient-specific templating for total knee arthroplasty

**DOI:** 10.1038/s41598-024-77794-9

**Published:** 2025-01-10

**Authors:** Mahmoud A. Hafez, Arne Jansen, Frank Portheine, Branislav Jaramaz

**Affiliations:** 1 The Orthopaedic Department, Faculty of Medicine, October 6 University, Giza, Egypt; 2 Helmholtz-Institute of Biomedical Engineering and SurgiTaix, Aachen, Germany; 3 Robotics Institute, Carnegie Mellon University, 5000 Forbes Avenue, Pittsburgh, PA 15213 USA

**Keywords:** Total knee arthroplasty, Total knee replacement, Patient-specific templating, Cutting guides, Custom-made, Musculoskeletal system, Translational research

## Abstract

Patient-specific templating (PST), which is a sister procedure to patient-specific instrumentation (PSI) but hospital-based, is relatively less complex and less expensive than robotics and navigation. However, there are some concerns about the PST including the process of preoperative planning, 3D printing and material, positioning of PST intraoperatively, availability, and clinical value. The purpose of this study was to validate the technical accuracy and reliability of the PST technique in the lab and to report the outcomes of clinical application. To test the reliability of the PST technique, five observers positioned the PST templates five times over the distal femur and proximal tibial whilst a navigation system was used to measure the level of bone cutting, coronal and sagittal alignment, and rotation in both femur and tibia. The mean alignment error in all planes was 0.67° (maximum 2.5°). Concerning the bone (femoral and tibial) cutting, the mean error was 0.32 mm (maximum 1 mm). The qualitative and quantitative analysis showed an overall agreement between observers (p < 0.05). The laboratory part of this study showed that the positioning of the PST over the proximal tibia and distal femur during TKA is reliable. There were statistically insignificant intraobserver and interobserver variations.

## Introduction

The conventional technique of total knee arthroplasty (TKA) relies on a fruitful of instruments that have limitations in accuracy and reproducibility. Navigation and robotics have been introduced to improve accuracy and reproducibility. However, broad-based application is limited due to high cost and complexity^1^.

Patient-specific templating (PST) is an alternative technique that can completely replace conventional instrumentation systems^2,3^. The CT-based preoperative planning was used to produce cutting blocks for TKA allowing the surgeon to perform the whole procedure without resorting to intra- or extra-medullary guides, driving down the number of instruments, steps, and costs^4^.

The technical steps for the PST technique include CT scanning, reconstruction of 3D images, sizing and alignment of prosthetic components, template designing, surgical simulation, and the production of PST using a rapid prototyping machine.

The reported accuracy of PST had errors within three degrees, which could be acceptable, provided that good positioning of the templates on the bone during surgery is achieved^2,3^. This positioning is based on surface matching between the templates and the respective bones (distal femur and proximal tibial). PST has a risk of malpositioning intraoperatively, which would result in errors in bone cutting, implant placement, and overall limb alignment. This risk is higher for new users, and this was not tested in the initial study^3^.

The principles of PST have been employed and commercialized by major implant companies; several versions of patient-specific instruments (PSI) or what is called custom-made cutting blocks, and pin-locator guides were released and have been in use. A comparison between the classic PST technique and different versions of PSI from different implant companies has been previously reported^5^.

Several review articles have been published looking at the functional outcome, efficacy, and accuracy of alignment with comparison to conventional instruments and other computer-assisted techniques^6–16^. However, all these publications did not apply to the classic technique of PST as the commercially available PSI techniques are produced by implant companies. Unfortunately, they are confined to developed countries due to their high costs. These techniques have major limitations that limit the broad-based application including cost, availability, long waiting time, complexity of imaging protocols, and confinement to straightforward cases of TKA^4^. Therefore, there is a need to report the accuracy and clinical outcome of the classic PST technique which is available in developing countries.

The purpose of this study was to validate the accuracy and reliability of the PST technique, particularly the positioning of the femoral and tibial templates.

## Methods

**Fig. 1:**
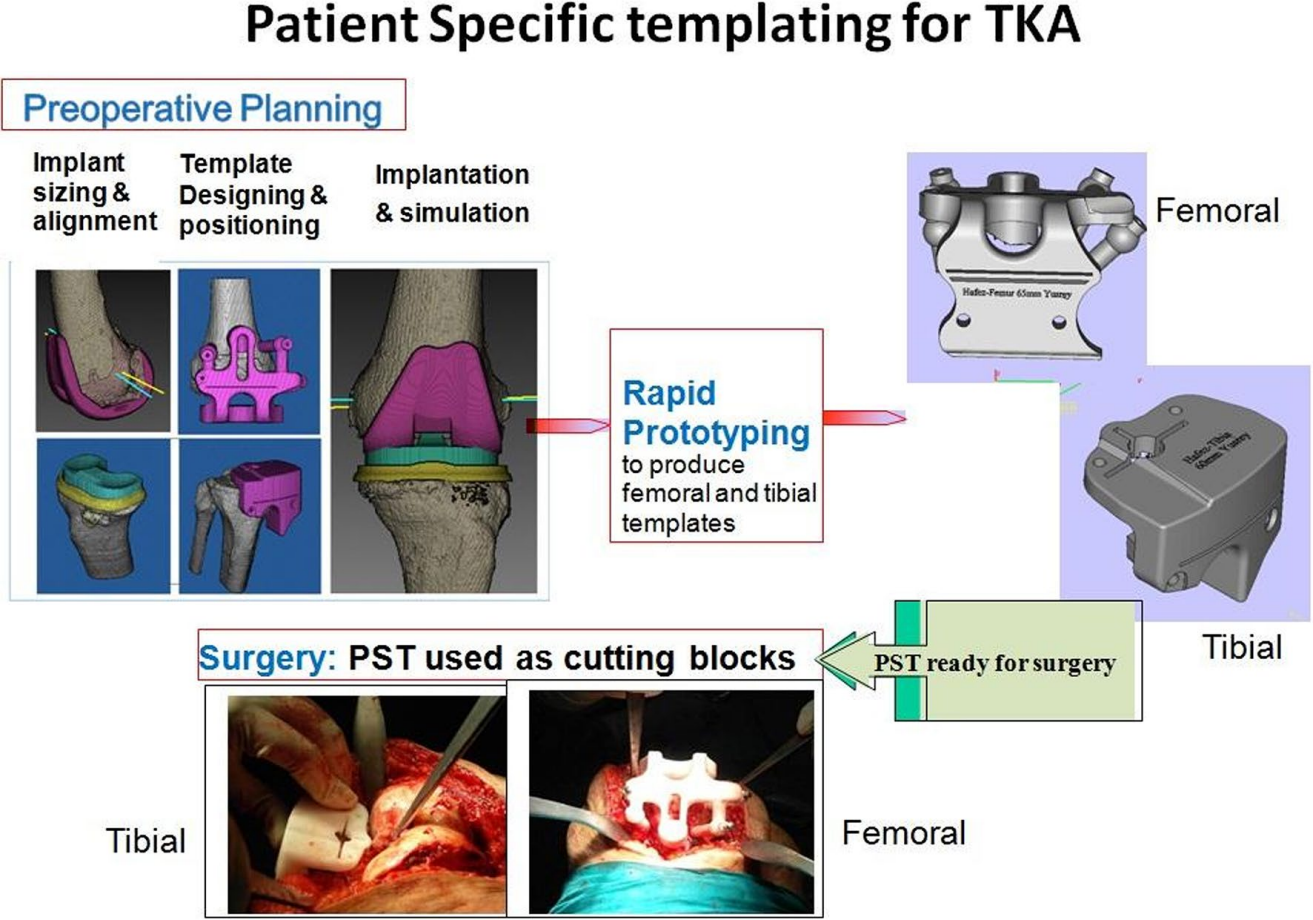
Typical steps used for computer-assisted patient-specific templating for total knee arthroplasty.

Five observers conducted a laboratory experiment: four independent observers who were new to the PST technique and the first author, who was the developer. A plastic knee specimen (Foam Cortical Shell, Model No. 1151) was used in the experiment. The PFC prosthesis (DePuy/Johnson and Johnson, Warsaw, IN, USA) served as the basis for the TKA planning, and the planning system incorporated electronic implant data. To fabricate PST, planning was completed, and the digital designs were submitted to a 3D printer (3D Systems, Valencia, CA, USA) (Fig. 1). The alignment and leveling of the bone cutting, shown by the templates’ positions, served as the main outcome measure.

The PST technique is based on having two templates, one for the femur and one for the tibia, to be used as cutting blocks. These templates feature slits allowing for five flat bone cuts in the distal femur and one cut in the proximal tibia. They are equipped with cylindrical locators, five for the femur and four for the tibia, positioned internally to match the contours of the respective bones. These patient-specific locators ensure precise and secure placement of the templates. Also, cannulated locators permit the insertion of fixation pins, enhancing stability over the bone. Additionally, lug holes/openings are included for stem fixation and keel placement^3^.

The observers did not utilize the navigation system (VectorVision, BrainLab, Heimstetten, Germany) to guide them; instead, it served as a measuring tool for the template location (the observers were not facing the navigation monitor when positioning the templates). However, the BrainLab navigation system was used for validation^17,18^.

The following were the standard procedures for utilizing navigational systems in TKA (in our case, using Sawbones): Anatomical and kinematic data, including significant landmarks like the epicondylar axis and the centers of the hip, knee, and ankle, were gathered after two tracking pins were placed into the proximal tibia and the distal femur, respectively, at a handbreadth from the knee joint (passive optical tracking was utilized). By tracking a tracking plate that was inserted one at a time into the slits of the femoral and tibial templates that were made for all reference cuts, the navigation system was able to use all the data to create a model that was unique to the plastic specimen and enable the measurement of alignment, rotation, and level of bone cutting in real-time. To monitor alignment and amount of bone cutting before performing real bone cuts, this phase was usually carried out during guided total knee arthroplasty (TKA). The tracking plate was put into the tibial, distal femoral, and anterior femoral slits of the traditional cutting blocks.

**Fig. 2:**
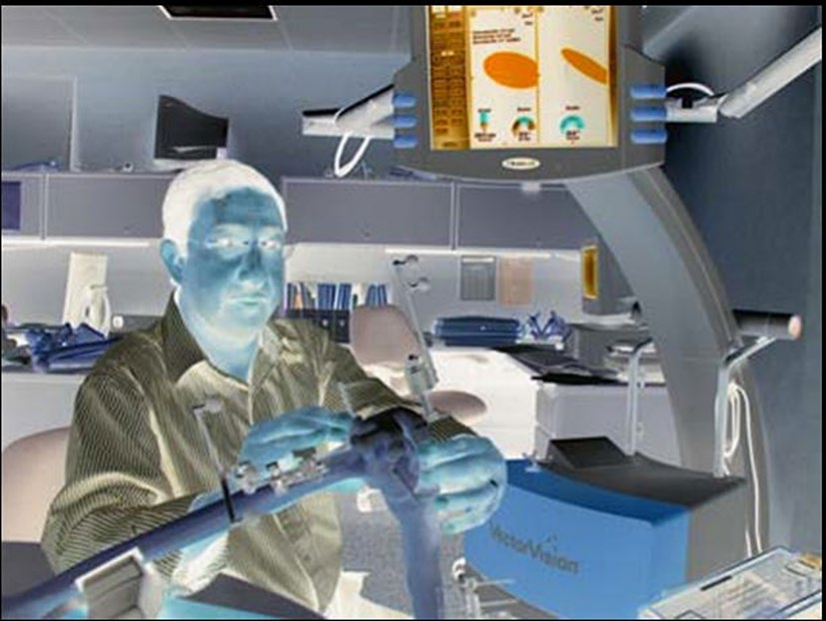
The lab setting with the first author placing the tibial templates while keeping the monitoring plate in place.

One by one, each observer was instructed to place the tibial templates while keeping the monitoring plate in place (Fig. 2). The tracking plates’ location was continually monitored by the navigation system, which then measured the alignment (coronal and sagittal) and degree of bone cutting. The results were shown in real-time on a computer monitor. An impartial assessor noted the measurements shown on the navigation monitor after the observer was happy with the template location. These measurements were taken before bone grafting, using a procedure akin to standard navigational TKA. For the femoral template location, the identical procedure was carried out five times each by five observers. There were two reference cuts made in this instance, though, and the templates were moved twice, the first time with the tracking plate placed in the distal femoral slit and the second time with the tracking plate placed in the anterior femoral slit. The anterior slit was used to quantify femoral rotation, whereas the distal femoral slit was used to measure alignment (coronal and sagittal) and the degree of bone cutting.

### Measurements

For each of these seven measurements—tibial coronal alignment, tibial sagittal alignment, level of tibial cutting, femoral coronal alignment, femoral sagittal alignment, femoral rotation, and level of femoral cutting—175 observations—25 sets of observations (5 observers × 5 times)—were to be collected. Apart from femoral rotation, which had only 13 sets of data rather than 25, it was only feasible to gather 163 observations or 25 full sets of observations for all measured parameters. This occurred during the experiment when the navigation system abruptly halted, maybe because of using the equipment for an extended period. Nevertheless, because it was challenging to precisely determine the angle of rotation based on vague cues, such as the medial third of the tibial tuberosity, tibial rotation was not evaluated in this experiment.

The standard alignment parameters—rotation and degree of bone cutting—that are also adhered to the preoperative PST planning were compared to these measured values. The amount of bone cutting in both the femur and tibia was 10 mm from the healthy component. The femoral and tibial coronal cuts were 0° to the mechanical axis, the femoral sagittal cut was in 3° flexion, and the tibial sagittal cut was 5° in the posterior slope. The variation of recorded values in degrees and millimeters was calculated using these suggested figures as control measurements (ground truth). Error was defined as the discrepancy between the recorded and control measures. When the recorded measurement was equal to the control measurement, the error was considered zero. These errors were analyzed to calculate the mean, standard deviation, and maximum (outliers).

### Statistical analysis

Both qualitative and quantitative data were utilized to quantify intraobserver and interobserver agreement to evaluate the PST technique’s dependability. To assess whether the recorded measurement was higher than 3° or 3 mm (no agreement) or less than 3° or 3 mm (agreement), kappa statistics were utilized to examine the qualitative data. The use of 3° as a limit was based on clinical studies that showed 3° to be the maximum error that could be clinically accepted^19^. Kruskal Wallis analysis of variance and Fredman’s repeated measure nonparametric analysis of variance (ANOVA) were used for the quantitative analysis. Every observer’s measured data was compared to a control measurement of zero mm or zero°. The perfect measurement, or one with no error, is represented by this control. The Kundall coefficient of concordance was used to measure the concordance between and within observers. The Pearson moment correlation test (r) was used to correlate the study observers’ data. A P value less than 0.05 was considered significant. All statistical calculations were done using both Microsoft Excel and Statistical Package for the Social Science (SPSS) statistical program. The mean standard deviation and maximum errors were calculated separately for each alignment (tibial coronal, tibial post slope, femoral coronal, and femoral sagittal) and each bone cutting level (femoral distal cut and tibial cut).

## Results

Laboratory results showed that the positioning of the PST and the alignment of the subsequent femoral and tibial bone cuts had a mean error of 0.67°. Breaking it down, the mean angle errors of the tibial coronal, tibial post slope, femoral coronal, and femoral sagittal were 0.36° (SD = 0.12), 1.24° (SD = 0.25), 0.90° (SD = 0.18), and 0.18° (SD = 0.01), respectively. The maximum error was 2.5°, which was recorded for the posterior sloping of one of the tibial cuts for one observer. The mean error in positioning the templates for the level of bone cutting was 0.32 mm (maximum 1 mm), 0.38 mm (SD = 0.10) for the femoral distal cut, and 0.26 mm (SD = 0.12) for the tibial cut. These measurements were calculated by our navigation system (Table 1).

**Table 1:** The reliability test: Errors and interobserver agreement.

Alignment and bone cutting errors	Tibial coronal (degrees)	Tibial post slope (degrees)	Femoral coronal (degrees)	Femoral sagittal (degrees)	Femoral distal cut (mm)	Tibial cut (mm)
Mean	0.36	1.24	0.90	0.18	0.38	0.26
Standard deviation	0.12	0.25	0.18	0.01	0.10	0.12
Range	0 to 0.50	0.5 to 2.50	0 to 1.50	0 to 0.40	0 to 1.00	0 to 0.50
Interobserver agreement (P-value)	< 0.003	< 0.014	< 0.002	0.002	< 0.039	< 0.002
Interobserver agreement (Concordance coefficient)	0.800	0.617	0.814	0.812	0.502	0.840

All measured values were within 3°, indicating complete interobserver and intraobserver agreement. For quantitative analysis, the Friedman test and Kendall concordance coefficient were used and an overall significant agreement between the observers was recorded (p < 0.05). A considerable interobserver agreement was perceived, proven by the high concordance coefficient for all measured parameters except femoral cutting level which showed a relatively low concordance coefficient (Table 1). A comparison between different recorded measurements for the same observer (intraobserver variation test) showed significant agreement (p < 0.003), and the concordance coefficient was very high. This means there was no difference after repeating the same test by the same observer and there was a considerable intraobserver agreement (Table 2).

**Table 2:** The reliability test: Intraobserver agreement.

Observers	A	B	C	D	E
Intraobserver agreement (P-value)	< 0.001	< 0.001	< 0.001	< 0.001	0.003
Concordance coefficient	0.9497	0.9278	0.9638	0.9662	0.7118

Based on the 13 observations for femoral rotation measurements, the mean error of rotation was 1.86° (the standard deviation was 0.02° and the maximum error was 2.84° of excessive external rotation), as compared to the preoperative planning of 3° external rotation. No analysis was performed to determine interobserver or intraobserver variation for femoral rotation because of the incomplete data and the reduced number of observers (only 3 out of 5 observers).

## Discussion

**Fig. 3:**
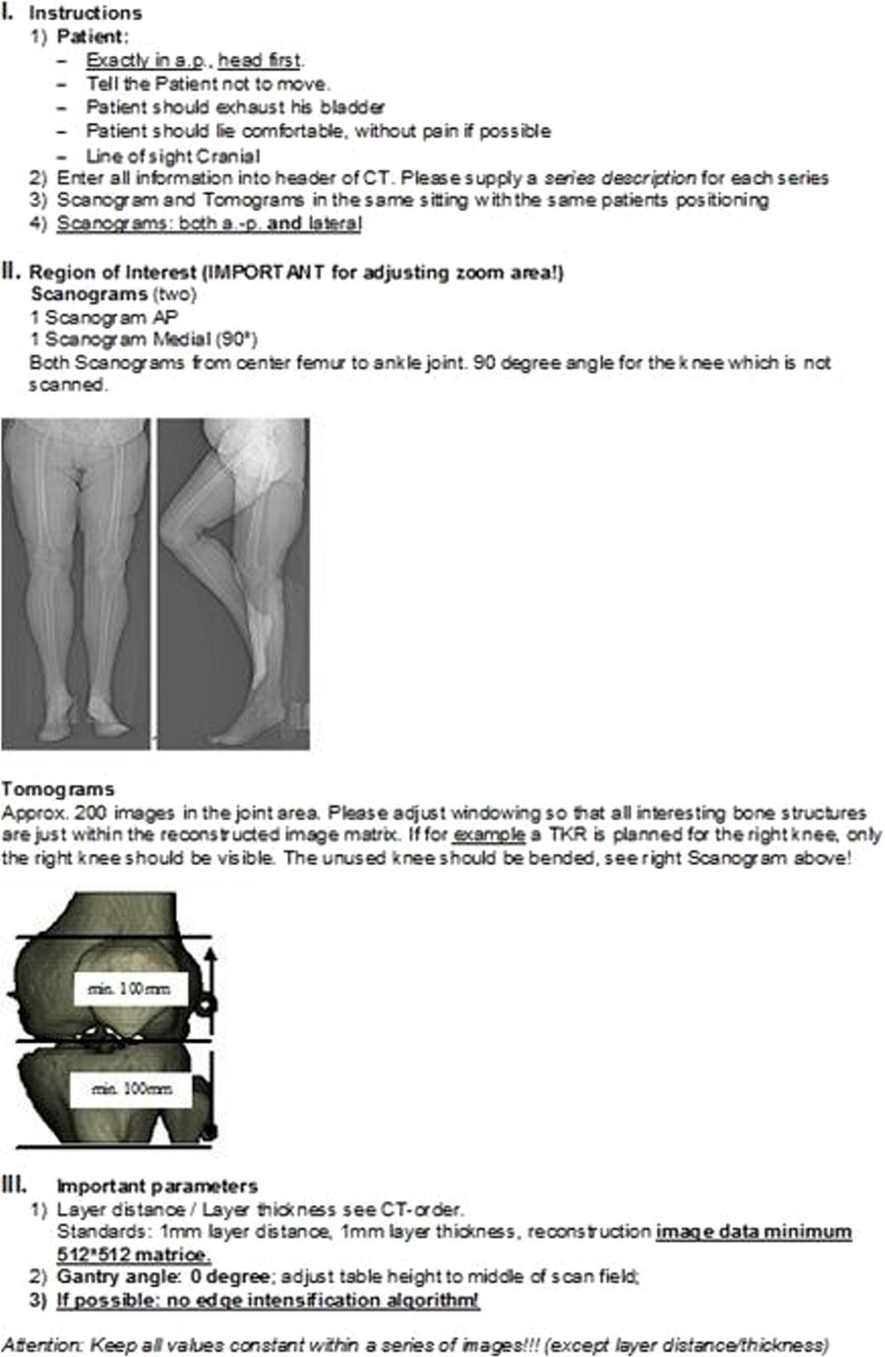
A summary of the planning including sizing and positioning of the implants.

In 2008, the PST technique was introduced for clinical application in a fail-safe manner. Conventional instruments were used to assist the accuracy of PST and to guide the bone cutting similarly to the laboratory study, while a navigation system was used to assess the accuracy of positioning the PST. This makes the surgeon more comfortable to proceed with such clinical applications. A summary of the planning including sizing and positioning of the implants was provided to the surgeon preoperatively in the form of screenshots (Fig. 3). These screenshots helped the surgeon position the template over the bone .

Theoretically speaking, PSI produced by implant companies have five advantages over conventional techniques: 1) reduced operative time; 2) fewer complications including less blood loss, transfusion, and fat embolism; 3) preferred by surgeons, anesthetists, nurses, and managers as compared to robotics and navigation; 4) a smaller number of assistants; and 5) more suitable for EADs and retained metalware. Still, its practical use and cost-effectiveness are not proven because it has five drawbacks: 1) it is an additional tool because it acts as pin locator and does not replace conventional instruments; 2) conducting MRI is expensive, not suitable for all patients, difficult to segment, and time-consuming; 3) planning is done by technician, that is why it is confined to straightforward cases; 4) it takes 4 to 6 weeks to deliver because of outsourcing; and 5) PSIs are expensive, around US$1000.

We believe the classic PST technique has the following advantages over implant PSI techniques. These advantages are: 1) most suited for bilateral simultaneous cases of TKA especially with severe intra- or extra-articular deformities; 2) ability to do difficult and complex cases, e.g., medically unfit, bilateral hemophilia; 3) short delivery time when using desktop 3D printers, 5) cheaper than PSI that are produced by implant companies; and 6) it acts as cutting blocks, so it eliminates conventional instruments and its drawbacks such as repeated sterilization, inventory, cost, slow learning, increased infection risk, … etc.^20–22^.

A comparison between the PST and PSI produced by implant companies, with regards to the advantages and pitfalls, was reported by two surgeons who are users of these two different techniques. They discussed different parameters including the cost, scanning, time, surgical learning curve, verification, accuracy, patellar kinematics, and soft tissue balancing^23^.

Klatt et al. revealed suboptimal clinical results of PSI (mechanical axis > 3°) when an image-free navigation system was used to evaluate the recommended custom-made cuts and alignment of the components^24^. Also, a meta-analysis by Thienpont et al. reported that the risk for tibial sagittal-plane malalignment was higher in PSI when compared to conventional instrumentation^8^. These results confirm the rationale and the clinical relevance of our study which was meant to validate this technique and test the reliability of template positioning.

There are several studies on clinical outcomes and alignment accuracy of PSI. However, the literature is lacking laboratory validation of these PSI techniques. In this work, the classic PST showed no significant intraobserver/interobserver variations for alignment or levels of bone cutting in both the femur and the tibia. This is comparable with the results of a postoperative CT scan that was done for the PST technique in a previous study^3^, where the mean errors for alignment and bone cutting were within 1.7° and 0.8 mm, and maximum errors were less than 2.3° or 1.2 mm. The procedure was not only limited to bone cuts done by PST but also included all other steps such as the preparation of lug holes, stem, and keel.

There are also secondary studies (meta-analysis and systematic reviews) on PSI techniques. Kizaki et al. found that using PSI in TKA did not improve the surgery time, complication rate, or patient-reported outcomes when compared to the standard conventional techniques. Also, despite being able to reduce blood loss, it was not efficient enough to reduce the transfusion rate. This was also verified by another meta-analysis which has found that PSI has resulted in less blood loss and shorter operative time^6,8,14^.

Mannan et al. reported there is no evidence to either support or discourage the use of PSI against the standard technique concerning the short-term functional outcomes. He strongly urged the need for high-quality studies comparing both techniques regarding the mid- and long-term outcomes^7^. Ren et al. discouraged the use of PSI in primary TKA. He has reported that PSI is not superior to conventional technique in the knees without any varus/valgus deformities, contractures, or bone loss^9^. Another study done by Woon et al. found no superiority of Kinematic alignment (KA) using PSI over mechanical alignment with respect to the patient-reported outcomes. The inaccuracy of the PSI used in KA could affect the outcome^11^.

An et al. reported that patient-specific guides performed using MRI had a lower proportion of outliers in the coronal alignment when compared to CT-based guides, but with no significant difference between them in terms of femoral and tibial component placement. Further studies are needed to guide the surgeon’s decision on making the ideal choice for imaging modality^10^. The same results were confirmed by another two studies later in the same year^12,13^. Lin et al. reported that MRI-based PSI reduces operative time and malalignment of the mechanical axis compared to CT-based PSI. Moreover, the Visionaire-specific PSI has better alignment results of the mechanical axis than other systems^16^. Thienpont et al. indicated that PSI improves the accuracy of both the femoral component alignment and global mechanical alignment, yet the tibial alignment remains to be verified^8^.

Recently, a systematic review by Confalonieri et al. reported that despite computer-assisted surgery (CAS) has proved to be a useful tool to achieve more accurate results of postoperative mechanical axis and higher functional scores, no meta-analysis has proved that PSI improves the mechanical axis or implant survivorship^15^. In addition, another study found that following TKA utilizing the PST approach, there was a considerable and virtually equal functional improvement in both groups. The rate of total problems was greater in the intra-articular group, though^25^.

### Strengths and limitations

This study has some limitations that need to be mentioned. Firstly, the measurements for femoral rotation were only done for three observers and were not complete as explained in the methods section. Measurements were also done before rather than after bone cutting. This had the advantage of eliminating errors, which are surgeon-dependent, rather than template-dependent. However, our study tested the intraobserver/interobserver variability which has not been reported earlier for other similar PSI techniques. Intraoperative measurements using the current navigation techniques are routinely performed before bone cutting, as the cutting process itself cannot be navigated due to the vibration of the saw blades. Lastly, in this paper, we only assessed the positioning of the cutting blocks on synthetic, not real bone. While this may provide some information regarding the laboratory validation of the PST technique, the overall accuracy and applicability (e.g., block pinning, bone cutting, cut verification) were not assessed.

## Conclusion

The laboratory validation of the PST technique showed a satisfactory level of accuracy and reliability of the technique for TKA. However, PST has its drawbacks, as it requires CT scans, which are not a routine requirement for TKA. Unlike navigation, templating techniques do not normally provide intraoperative measurements, since sizing, alignment, and bone cutting are determined preoperatively^26^. Also, PST has the potential to be used as a training tool, allowing complete planning of surgery with a 3D simulation that facilitates the identification and correction of errors in real time. Moreover, it allows the measurement of surgical performance by comparing postoperative imaging with recorded preoperative planning. Further, templating techniques also have the potential to reduce the risk of infection, by shortening the operative time and eliminating medullary perforation, excessive bleeding, and tracking pins (in the case of navigation techniques). Since PST provides single-use instruments, it may be useful in areas where there is a high risk of variant Creutzfeldt-Jakob disease (vCJD) that requires an extraordinarily high level of sterilization. Lastly, we would like to highlight that the first author worked on a modification to the software and 3D printing techniques to allow the application of a new concept called “open platform”^27^, which allowed the use of PST for any TKA implants without limitations.

## Data Availability

All data generated or analyzed during this study are included in this published article.
